# Verbal/social autopsy analysis of causes and determinants of under-5 mortality in Tanzania from 2010 to 2016

**DOI:** 10.7189/jogh.10.020901

**Published:** 2020-12

**Authors:** Alain K Koffi, Henry D Kalter, Mlemba A Kamwe, Robert E Black

**Affiliations:** 1 Institute for International Programs, Department of International Health, Bloomberg School of Public Health, Baltimore, Maryland, USA; 2 National Bureau of Statistics, Dodoma, Tanzania

## Abstract

**Background:**

Tanzania has decreased its child mortality rate by more than 70 percent in the last three decades and is striving to develop a nationally-representative sample registration system with verbal autopsy to help focus health policies and programs toward further reduction. As an interim measure, a verbal and social autopsy study was conducted to provide vital information on the causes and social determinants of neonatal and child deaths.

**Methods:**

Causes of neonatal and 1-59 month-old deaths identified by the 2015-16 Tanzania Demographic and Health Survey were assessed using the expert algorithm verbal autopsy method. The social autopsy examined prevalence of key household, community and health system indicators of preventive and curative care provided along the continuum of care and Pathway to Survival models. Careseeking for neonates and 1-59 month-olds was compared, and tests of associations of age and cause of death to careseeking indicators and place of death were conducted.

**Results:**

The most common causes of death of 228 neonates and 351 1-59 month-olds, respectively, were severe infection, intrapartum related events and preterm delivery, and pneumonia, diarrhea and malaria. Coverage of early initiation of breastfeeding (24%), hygienic cord care (29%), and full immunization of 12-59 month-olds (33%) was problematic. Most (88.8%) neonates died in the first week, including 44.3% in their birth facility before leaving. Formal care was sought for just 41.9% of newborns whose illness started at home and was delayed by 5.3 days for 1-59 month-olds who sought informal care. Care was less likely to be sought for the youngest neonates and infants and severely ill children. Although 70.3% of 233 under-5 year-olds were moderately or severely ill on discharge from their first provider, only 29.0%-31.2% were referred.

**Conclusions:**

The study highlights needed actions to complete Tanzania’s child survival agenda. Low levels of some preventive interventions need to be addressed. The high rate of facility births and neonatal deaths requires strengthening of institutionally-based interventions targeting maternal labor and delivery complications and neonatal causes of death. Scale-up of Integrated Community Case Management should be considered to strengthen careseeking for the youngest newborns, infants and severely ill children and referral practices at first level facilities.

Reduction of child mortality is an important global goal adopted by many countries that requires information on the levels and causes of death [1]. In the absence of a strong vital registration or population-based surveillance system, data on child mortality levels have come from national demographic surveys [2]. In these settings, information on causes of death largely comes from “verbal autopsies”, post-mortem interviews of the deceased children’s caregivers to inquire about signs and symptoms prior to death [3]. Ideally these assessments of causes are done with representative samples of child deaths, such as those identified in national surveys. These interviews can also include a “social autopsy” with questions about the response of the family and health system to the child’s fatal illness.

Tanzania has achieved more than two-thirds reduction in its under-five mortality rate, from 166 deaths per 1000 live births in 1990 to 53 in 2018 [2]. The country does not have sufficient registration of child deaths and medical certification of the causes and has relied on periodic national surveys for trends in mortality rates. Previous estimates of the causes of death have been modeled using global data linked to national covariates [3,4]. We conducted a nationally representative verbal autopsy and social autopsy (VASA) study in Tanzania to directly estimate the cause of death distributions and the prevalence of social and health system determinants of death, separately for neonates (0-27 days old) and young children (1-59 months old).

## METHODS

Data for this paper come from the VASA study, which was conducted on the platform of the 2015-16 Tanzania Demographic and Health Survey (TDHS) of 13 360 households [5]. The TDHS included a lifetime birth history of all women 15-49 years old to identify all live births and deaths of these children. In addition to the supplemental pregnancy calendar filled for all DHSs, the TDHS birth history was followed by specific questions on all births in 2010-2016 and those that did not end in a live birth were entered in the calendar as pregnancy terminations. Pregnancy terminations of seven or more months were considered to be stillbirths.

Following completion of the TDHS, VASA interviews were conducted of all 851 7-plus-months pregnancy terminations and all under-5 deaths in the five years prior to their TDHS interview. Because the TDHS was conducted from August 2015 to February 2016, the VASA study deaths encompassed the five and one-half year period from August 2010 to February 2016. The VASA questionnaire consisted of the original Population Health Metrics Research Consortium (PHMRC) verbal autopsy questionnaire [6] chronologically integrated with the updated Maternal and Child Epidemiology Estimation group (MCEE, formerly known as the Child Health Epidemiology Reference Group (CHERG)) social autopsy questionnaire [7] to ask about all illness events in the order in which they occurred. The questionnaire was translated from the original English to Kiswahili, independently back-translated and consistency checked by personnel from the study’s implementing agency, the Tanzania National Bureau of Statistics (NBS). A local social scientist checked the questionnaire and conducted key informant interviews to ensure use of local terminology understood by lay respondents. The questionnaire was entered into CSPro-X software and the interview conducted on netbook computers using a CSPro Computer Assisted Personal Interview (CAPI) software application designed to guide and assist data collectors through the interview with a minimum of data capture errors.

The interviews were conducted nationwide, overseen on the mainland by NBS and in Zanzibar by the Office of the Chief Government Statistician (OCGS). The data collectors were all females with at least a secondary education and experienced in conducting demographic and health interviews. Training consisted of a 2-week training of NBS and OCGS trainers, who then led a 3-week training of interviewers and supervisors, including classroom didactic presentations, interview practice and role plays, and field practice interviews with mothers of recently deceased children. Fieldwork was conducted from mid-November through December 2017, with a second round from mid-January to mid-February 2018 to locate and interview respondents who had moved from their original location. Throughout both rounds, data quality was ensured by the technical team/trainers supported by the Johns Hopkins University team.

### Birth status and cause of death analysis

The VASA interview first evaluated possible TDHS misclassification of 7-plus-months pregnancy terminations (assumed by the TDHS to be stillbirths) and deaths of live born children by asking about cardinal signs of life at birth not asked about by the TDHS, including any breathing, crying or movement. A child was considered stillborn if reported to have never cried, even a little bit, never moved, and never breathed, even a little. If one or more positive signs were present at birth, then the child was considered to have been born alive and was classified as a neonatal or 1-59 month-old death, depending on the VASA-reported age at death. The current analysis is of neonatal and 1-59 month-old deaths, including deaths of live born children misclassified by the TDHS as stillbirths and excluding stillbirths misclassified by the TDHS as child deaths.

Causes of death were assessed using the expert algorithm verbal autopsy (EAVA) method, with algorithms of illness signs and symptoms arranged in a hierarchy to select the underlying cause. The EAVA method for neonatal and 1-59 month-old deaths has been previously described [8] and validated [9]. In brief, algorithms of causes of death of public health importance were formed based on prior VA validation studies, consultation with VA experts and a literature review of associated illness signs and symptoms. Separate hierarchies of the neonatal and 1-59 month-old death causes were derived to select the underlying cause of death according to International Classifications of Diseases, 10th Revision (ICD-10) rules, illness temporality and specificity. For example, meningitis is above sepsis in the neonatal hierarchy because it refers to the source of infection. Although not in agreement with ICD-10 rule P1 that the mode of perinatal death, including prematurity, should not be classified as the main disease or condition unless it was the only condition known, preterm delivery is near the top of the neonatal hierarchy based on this placement’s superior performance in a validation study [9].

The 1-59 month-old hierarchy includes three ‘neonatal’ conditions (congenital, intrapartum related events, or IPRE, and preterm delivery) that started during the first month of life but resulted in death from months 1-11. Preterm is considered as a contributing or risk factor for child deaths, so is placed at the bottom of the hierarchy, while IPRE and congenital are placed at the top under the assumption that children with these conditions survived to the post-neonatal period through intensive care or surgery and later succumbed to their underlying neonatal condition. Several conditions in both hierarchies, including neonatal diarrhea and pneumonia and 1-59 month-old dysentery, diarrhea, pneumonia and malaria, have ‘probable,’ more specific, algorithms higher up in the hierarchy and ‘possible,’ more sensitive, algorithms lower down. Probable and possible deaths from these causes are combined for their final counts. The **Online Supplementary Document** provides the algorithms and hierarchies utilized for this study.

### Determinants of death (social autopsy) analysis

The social autopsy analysis was descriptive in nature, and entailed examining the prevalence of key household, community and health system indicators of preventive and curative care provided along the continuum of newborn and child care and the Pathway to Survival models [7,10,11], respectively. A list and definitions of some of the operational variables used throughout this paper are available in articles by Koffi et al. [12]. Illness severity was ranked as mild, moderate or severe at three points along the Pathway, including when the illness was first noticed, when the decision was taken to seek formal health care, and when leaving the first and last formal providers. The severity rank was based on signs previously shown to be noted by mothers in association with severe neonatal illness [12], including the child’s feeding behavior (normal, poor, none), level of consciousness (alert, drowsy, unconscious) and activity level (normal, less than normal, not moving).

### Statistical analyses

The CSPro data collected on netbooks were converted to SAS 9.4 [13] and STATA 15.0 [14] data sets for analysis. Following determination of the neonatal and 1-59 month-old deaths from the TDHS data set, all subsequent analyses of cause distributions and social determinants were conducted of data weighted on the basis of the TDHS multi-stage sampling design. Descriptive statistics used for the study included frequency distributions. Although the weighted analyses resulted in fractional frequencies, for simplicity sake this paper’s text rounds these up to the next higher whole number. Tests of association included odds ratio with 95% confidence interval, the independent samples *t* test that compares the difference in the means of the two groups, and corrected Mantel-Haenszel χ^2^ and *P* values associated with these tests.

### Ethical considerations

The VASA study in Tanzania was first approved by the National Institute for Medical Research (Tanzania Mainland), and the Ministry of Health (Zanzibar), then by the Johns Hopkins School of Public Health’s Institutional Review Board. All respondents provided informed consent before the interview was conducted.

## RESULTS

Of the 851 TDHS-identified stillbirths and under-5-year-olds deaths, 783 (92.0%) had a VASA interview completed, of which 204 were determined to be stillbirths, 228 neonatal deaths and 351 1-59 month-old deaths. The current analysis is of the neonatal and 1-59 month-old deaths. On average, it took 4.0 to 4.1 years (IQR = 3 years; 5 years) from the neonatal and child date of death to the VASA interview date, respectively. The child’s mother was the respondent for 91.1% and 89.7% of the neonatal and 1-59 month-old deaths, respectively.

### Age, sex and place of birth and death

Of 228 neonatal deaths, 104 (45.5%) died within 24 hours of birth, 203 (88.8%) died before completing the first week of life, and 216 (94.9%) died in the first 2 weeks. Of 351 1-59 month-old deaths, 157 (44.7%) died within their first year, and 252 (71.8%) died before age of 2 years. One hundred twenty-nine (56.4%) of the neonatal deaths were male and 99 (43.6%) were female. The sex ratio was reversed for the 1-59 month-old deaths, with 164 (46.7%) males and 187 (53.4%) females.

Altogether, 142 (62.5%) of 228 neonates died in a health facility. Of 111 (48.9%) hospital deaths, 76 (68.3%) were born and died in the same facility without leaving. The same was true of 25 (80.6%) of the 31 (13.5%) neonates who died in a lower level facility. Of the 74 (32.4%) neonates who died at home, 44 (59.2%) were born at home. The odds of having been born in any health facility for those who died in any facility was 13.7 (95% CI = 7.0-26.9, χ^2^ = 68.9, *P* < 0.001); and the odds of having been born at home for those who died at home was 11.8 (95% CI = 5.9-23.5, χ^2^ = 59.6, *P* < 0.001).

Fewer of the 1-59 month-olds than neonates died in a health facility, and contrary to the neonates there were only weak associations between their place of birth and death. Altogether, 168 (47.8%) of 351 children died in a health facility. Of 131 (37.2%) hospital deaths, 59 (45.3%) were born in a hospital and 29 (22.3%) in a lower level facility; and of 37 (10.7%) lower level facility deaths, 19 (51.6%) were born in a lower level facility and seven (19.5%) in a hospital. The odds of having been born in any health facility for those who died in any facility was 1.7 (95% CI = 1.1-2.6, χ^2^ = 5.1, *P* = 0.025). There was no association between dying at home and having been born at home (OR = 1.2, 95% CI = 0.78-1.93, χ^2^ = 0.8, *P* = 0.38).

### Demographic and household characteristics

**Table 1** also shows the characteristics of the mother, her partner/husband, and her household. Most mothers (79.5%) were married or cohabiting with a man at the time of their child’s death. Half the mothers first married by age 18 and 75% by age 20; and they were young when their child died, with a median age of 26 years. More than 8 in 10 mothers had some primary or higher level of education. Only 16% of households had electricity, 38% an inside or outside the house piped water supply and 22% used improved sanitation, such as a flush or improved pit toilet. Less than 2% of households used electricity or gas for cooking. On average, travel time from the household to the nearest health facility in an emergency was 30 minutes.

**Table 1:** Demographic characteristics of the deceased newborns and children

Characteristics	Total (under-five deaths) (N = 579)	Neonatal (0-27 d) deaths (N = 228)	Child (1-59 mo) deaths (N = 351)
Mother’s marital status:			
Married	365 (63.0%)	139 (64.4%)	226 (62.2%)
Living with a man	95 (16.5%)	35 (16.3%)	60 (16.6%)
Widowed	11 (2.0%)	3 (1.3%)	9 (2.4%)
Divorced, separated	66 (11.4%)	17 (8.0%)	49 (13.4%)
Never married/lived with a man	41 (7.2%)	22 (10.0%)	20 (5.5%)
Median age when first married	18; IQR = 17-20 (N = 518)	18; IQR = 17-21 (N = 204)	18; IQR = 17-20 (N = 314)
Maternal age at child death (years):			
<20	90 (15.6%)	44 (20.5%)	46 (12.6%)
20-24	146 (25.3%)	53 (24.6%)	93 (25.7%)
25-29	121 (21.0%)	47 (22.0%)	74 (20.3%)
≥30	175 (30.3%)	59 (27.1%)	117 (32.1%)
Missing/ Don't know	46 (8.0%)	12 (5.8%)	34 (9.3%)
Median age at child death	26; IQR = 21-32 (N = 539)	25; IQR = 20-31 (N = 217)	26; IQR = 22-33 (N = 322)
Maternal education:			
None	109 (18.8%)	28 (13.0%)	81 (22.2%)
Primary	417 (72.0%)	155 (72.1%)	261 (71.9%)
Secondary or more	53 (9.1%)	32 (14.9%)	21 (5.7%)
Don't know	1 (0.2%)	0 (0.0%)	1 (0.3%)
Median years of schooling	7; IQR = 4-7 (N = 578)	7; IQR = 6-7 (N = 228)	7 IQR = 3-7 (N = 350)
Paternal education:			
None	80 (13.8%)	23 (10.8%)	56 (15.5%)
Primary	352 (60.8%)	122 (56.6%)	230 (63.3%)
Secondary or more	71 (12.3%)	41 (19.2%)	30 (8.2%)
Don't know	76 (13.2%)	29 (13.4%)	47 (13.0%)
Median years of schooling	7; IQR = 6-7; (N = 508)	7.2/7; (N = 199)	7; IQR = 4-7 (N = 309)
Household:			
Mean/median household density (persons/rooms)	2.5/2.5; IQR = 1.75-3 (N = 576)	2.3/2; IQR = 1.5-3 (N = 227)	2.7/2.5; IQR = 2-3 (N = 349)
Main income: agricultural work	338 (58.3%)	126 (58.3%)	212 (58.3%)
Household has electricity	92 (16.0%)	43 (20.1%)	49 (13.5%)
Piped water inside or outside the house	219 (37.8%)	90 (41.5%)	130 (35.6%)
Improved sanitation	125 (21.6%)	49 (22.6%)	76 (21.0%)
Electricity or gas for cooking	9 (1.6%)	7 (3.3%)	2 (0.6%)
Median travel time to nearest health facility (minutes)	30; IQR = 15-60 (N = 578)	30; IQR = 15-60 (N = 227)	30; IQR = 15-60 (N = 351)

IQR – interquartile range

### Causes of death

**Figure 1:**
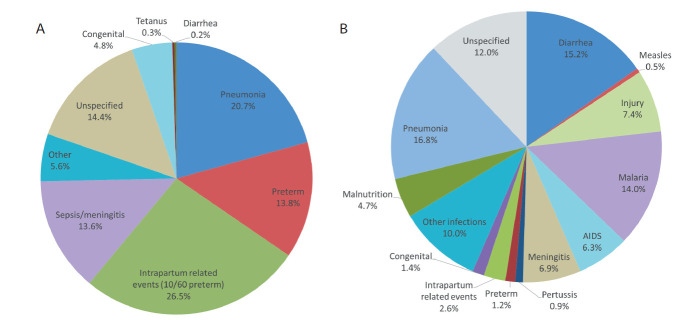
Expert algorithm verbal autopsy causes of death. **Panel A.** Expert algorithm verbal autopsy causes of death of 228 neonates. **Panel B.** Expert algorithm verbal autopsy causes of death of 351 1-59 month-olda, with two ‘neonatal’ causes (congenital and intrapartum-related events) at the top and one (preterm) at the bottom of the hierarchy that occurred during months 1-11.

**Figure 1**, Panel A shows the underlying causes of the 228 neonatal deaths. Intrapartum related events (IPRE: birth injury and, mainly, birth asphyxia), pneumonia and preterm delivery were the most common causes. It can be seen within the IPRE pie slice that another 10 (10/228 or 4.3%) neonates had co-morbid prematurity. This corresponds to the 40 neonatal deaths reported to have been the product of a 6- or 7-month gestation pregnancy, plus one additional death from respiratory distress syndrome (RDS) with 8-month gestation.

**Table 2** shows the distribution of the three most common causes of neonatal death, by age and place of death. Severe infection, including pneumonia, sepsis and meningitis, was most frequent, followed by IPRE and preterm. All IPRE, nearly all preterm, and four-fifths of severe infection deaths occurred during the first week. Nearly four-fifths of IPRE and two-thirds of early neonatal severe infection deaths occurred in a health facility, compared to less than half the early neonatal preterm deaths. Eighty-one (80.2%) and 1 (11.1%) of these early and late neonatal facility deaths, respectively, occurred in the child’s birth facility before discharge.

**Table 2:** Most common causes of neonatal death, by age and place of death

Cause of death	Early neonatal deaths		Late neonatal deaths		Total, N (%)
Facility N (%)*	**Community, N (%)**	**Facility, N (%)**	**Community, N (%)**
Severe infection†	41 (52.4)	22 (27.5)	6 (8.3)	9 (11.8)	78 (100)
Intrapartum-related events	47 (77.1)	13 (21.7)	1 (1.0)	0 (0.0)	61 (100)
Preterm delivery	13 (41.9)	16 (51.6)	2 (6.5)	0 (0.0)	31 (100)
**Total**	**101 (59.2)**	**51 (29.8)**	**9 (5.5)**	**9 (5.5)**	**170 (100)**

*Row percent.

†Severe infection: sepsis/meningitis and pneumonia.

**Figure 1**, Panel B displays the 1-59 month-old causes of death. Pneumonia, diarrhea and malaria were the most common causes. Other infections, meningitis and AIDS were also important, as were the non-infectious causes, injury and malnutrition. It can also be seen that 5.2% of the 1-59 month-old children died from one of the three neonatal causes.

**Figure 2:**
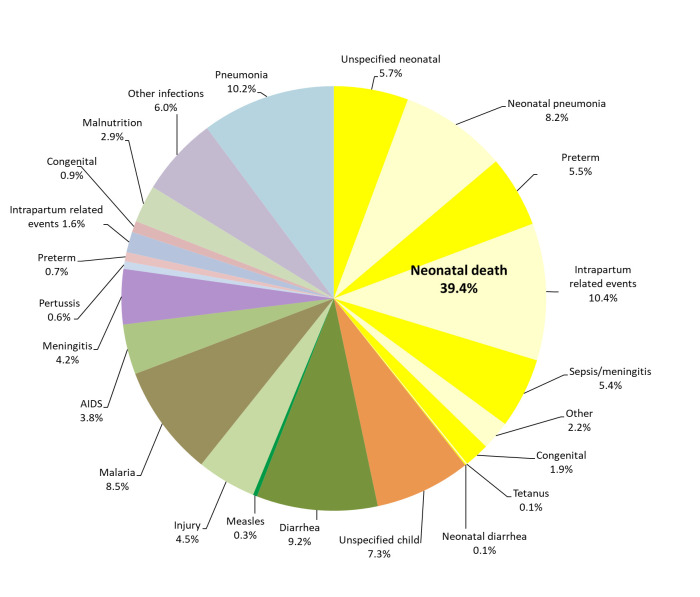
Expert algorithm verbal autopsy causes of death of 579 under-5 year-olds. Non-neonatal deaths from intrapartum-related events, congenital and preterm occurred during months 1-11.

**Figure 2** combines **Figure 1**, Panel A and Panel B, to provide the cause distribution of under-5-five deaths. The yellow pie slices represent the 39.4% of all under-5 deaths that occurred during the neonatal period. It can be seen that pneumonia was the most common cause, contributing 18.4% of all under-5 deaths. The second most common cause was IPRE, which caused 12.0% of under-5 deaths, including 1.6% during months 1-11, while diarrhea and malaria were nearly tied in third place with 9.2% and 8.5% of all deaths, respectively. Preterm delivery caused 6.2% of the under-5 deaths, with 0.7% of these occurring during months 1-11. Neonatal sepsis/meningitis caused 5.4% of all under-5 deaths, and child meningitis and AIDS were also important causes, with 4.2% and 3.8% of the deaths, respectively. Injury caused 4.5% of the under-5 deaths.

### Social autopsy determinants of death

**Figure 3:**
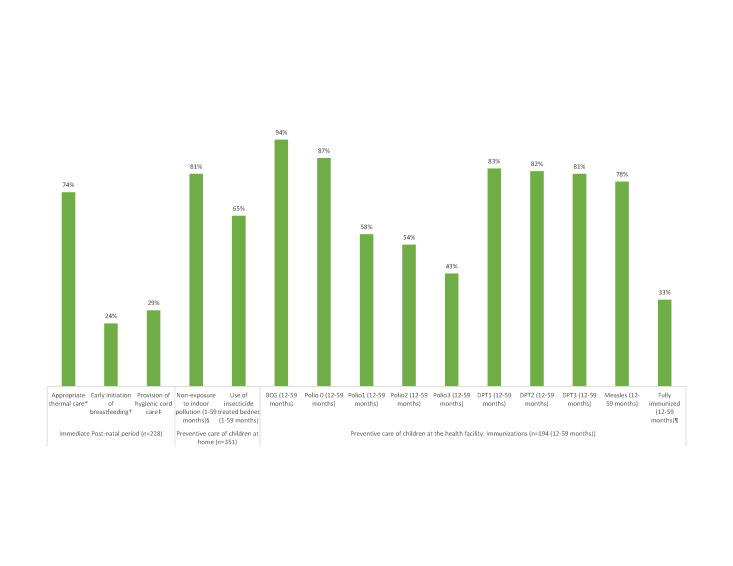
Coverage along the continuum of care for 228 neonatal and 351 child deaths. *Appropriate thermal (Among those who survived at least 24 hours) care consisting of immediate warming, drying and wiping, wrapping in a blanket, skin to skin contact with the mother or being placed in an incubator, plus bathing delayed for more than 24 hours after birth. †Early initiation of breastfeeding: within the first hour after delivery. ‡Provision of Hygienic cord care suggests a new boiled razor blade from the delivery kit was used for cutting the cord, a clean boiled piece of thread from the delivery kit was used for tying the cord and nothing was applied to the umbilical cord stump after birth or in case something was applied, either alcohol or other antiseptic or antibiotic ointment in cream or powder form was applied. §Proportion of children who were NOT usually beside or carried by their mother when she cooked inside the home. ¶Information on immunizations was obtained either from the vaccination card or when there was no written record, from the respondent (mainly the mother). Polio0 is the Polio vaccination given at birth; Fully Immunized children received BCG, measles, and three doses each of DPT and oral polio vaccine (excluding polio vaccine given at birth).

**Figure 3** shows the preventive care received by newborns and young children along the continuum of care. Overall, the coverage of most key interventions was relatively high. Yet, the coverage of two post-natal care interventions was problematic: early initiation of breastfeeding at 24% and hygienic cord care at 29%. Overall, only 33% of children were fully immunized against each of the six major preventable childhood diseases before they reached their first birthday. Coverage was highest for BCG, and lowest for OPV1-3, ranging from 43% to 58%. There was no significant difference in fully immunized status between male (25.3%) and female (39.7%) children (χ^2^ = 3.26; *P* = 0.07) at the 0.05 level.

**Figure 4:**
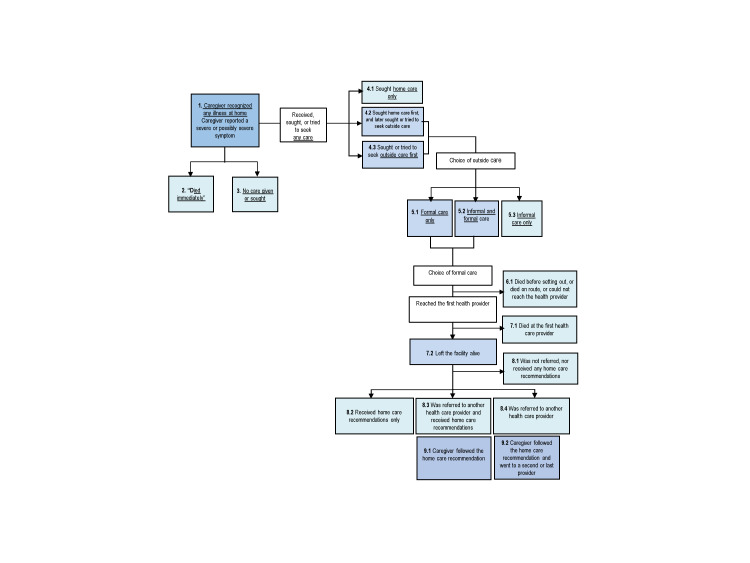
The “Pathway to Survival” Indicators.

**Figure 4** exhibits the steps where possible breakdowns in the Pathway to Survival may have contributed to deaths. **Table 3** depicts the distribution of these indicators for the neonates (N = 115) and 1-59 month-old children (N = 351). The 113 neonates whose fatal illness began at the health facility where they were delivered and either died without leaving (101) or left the health facility to a second provider (12) were excluded from this analysis, which was conducted to examine careseeking for sick children from home.

**Table 3:** Pathway to Survival Indicators of under-five deaths

Pathway to Survival Component and Indicators*	Under-five deaths	Neonatal deaths (0-27 d)	Child deaths (1-59 mo)	Χ-2 (*P*-Value)
**Illness recognition at home**	N = **466**	**N = 115**	**N = 351**	
1. Caregiver recognized sign(s) of possibly severe or severe illness	445 (95.6%)	105 (91.5%)	340 (96.8%)	**4.81 (0.029)**
**Care-seeking patterns**	**N = 464**	**N = 113**	**N = 351**	
2. Child “died immediately”	38 (8.3%)	23 (20.6%)	16 (4.6%)	**14.01 (0.002)**
3. No caregiven or sought for child	40 (8.6%)	34 (29.8%)	8 (2.3%)	**57.05 (0.000)**
4.1 Child received home care only	10 (2.1%)	6 (5.1%)	4 (1.2%)	3.46 (0.0639)
4.2 Child sought or tried to seek outside care as first action	308 (66.3%)	45 (40.1%)	260 (74.1%)	**26.24 (0.000)**
4.3 Child sought or tried to seek outside care as later action	68 (14.7%)	5 (4.3%)	63 (17.8%)	**7.76 (0.006)**
**Choice of outside care:**				
5.1 Formal care only	284 (61.2%)	47 (41.1%)	235 (67.0%)	**12.19 (0.001)**
5.2 Informal and formal care	70 (15.1%)	1 (0.8%)	68 (19.3%)	**36.52 (0.000)**
5.3 Informal care only	22 (5.9%)	3 (5.1%)	19 (6.0%)	**0.04 (0.8518)**
**Choice of any formal care:**	**n = 351**	**n = 48**	**n = 303**	
6.1 Child died before setting out, or died on route, or could not reach the health care provider	15 (4.2%)	3 (6.9%)	11 (3.8%)	0.73 (0.393)
7.1 Child reached the first health care provider and died at the facility	103 (29.0%)	26 (54.8%)	77 (25.3%)	**11.97 (0.001)**
7.2 Child reached the first health provider and left the facility alive	233 (66.8%)	18 (38.3%)	215 (70.9%)	**13.98 (0.000)**
**Decision of health provider at discharge**	**n = 233**	**n = 18**	**n = 215**	
8.1 child was not referred, nor received any home care recommendations	68 (29.3%)	3 (16.6%)	65 (30.4%)	1.888 (0.1724)
8.2 Child received home care recommendations only	93 (40.1%)	10 (54.5%)	83 (38.4%)	1.54 (0.2159)
8.3 Child was referred to another health care provider and received recommendations	39 (16.8%)	1 (4.60%)	38 (17.6%)	2.38 (0.1249)
8.4 Child was referred to another health care provider only	34 (14.2%)	5 (24.4%)	29 (13.6%)	1.18 (0.2780)
**The caregiver followed the recommendation and went to a second or last provider:**				
9.1 Caregiver followed all the home care recommendation	115/133 (86.5%)	10/11(69.4%)	105/121 (87.2%)	0.38 (0.535)
9.2 Referral compliance	55/72 (76.4%)	3/5 (57.2%)	52/67 (77.2%)	**9.88 (0.002)**

Several indicators of careseeking were significantly better for children than neonates. More caregivers of children (96.8%) than neonates (91.5%) recognized the presence of severe or possibly severe signs or symptoms at illness onset (χ^2^ = 4.81, *P* = 0.029). Formal care was sought or obtained for just 41.9% of newborns, compared to 86.3% of children (χ^2^ = 53.698, *P* < 0.001), respectively, 1.2 days and 2.8 days (*t* = -1.4857, *P* = 0.069) after the onset of the illness. However, the delay was 1.7 days among the 235 children who sought formal care only, compared to 7.0 days for the 68 who sought both informal and formal care (*t* = -5.17; *P* < 0.001).

Of the 48 newborns and 303 children who obtained formal care, mortality at the first provider reached was significantly higher (χ^2^ = 11.97 *P* = 0.001) for newborns (54.8%) than children (25.3%). Among those who left the facility alive, the referral rate was low for both age groups, 29.0% for newborns and 31.2% for children. This was despite the fact that 70.3% of the 233 neonates and children who left the provider alive were moderately to severely ill at discharge. Overall, 68 neonates and children were not referred nor received any home care recommendation, yet 67.0% of them had symptoms of moderate to severe illness. Of those who were referred, compliance was higher for children (77.2%) than neonates (57.2%) (χ^2^ = 9.88, *P* = 0.002).

In a unadjusted logistic regression model between care seeking and cause of death (**Table 4**), neonates who died from IPRE or prematurity were two-thirds less likely than neonates with other causes of death to receive any care inside or outside the home, although at borderline significance (OR = 0.32; 95% CI = 0.10, 1.12; *P* = 0.08). Likewise, newborns who died in the first week were 86% less likely than older neonates to receive any care (95% CI = 0.04, 0.53; *P* = 0.004) (data not shown). However, while early neonates and all neonates who died from IPRE or prematurity were also less likely to receive any care than other early neonates and all neonates, respectively, the difference was not significant when adjusted for age in days and perceived illness severity (**Table 4**). The only significant finding remaining was that the odds to have received any care increased by 15% for each additional day of age (AOR = 1.15; 95% CI = 1.04, 1.27; *P* = 0.008).

**Table 4:** Unadjusted and logistic regression adjusted analysis of factors possibly associated with careseeking from home for early, late and all neonatal deaths*

Age group: Explanatory factors	Died immediately/ Care not needed, given or sought	Care sought or tried to seek	Unadjusted Model, P-value, OR (95% CI)	Adjusted Model, P-value, AOR (95% CI)
**Early neonatal deaths (days 0-6)**				
Cause of death:				
-All other causes (reference)	27 (51.4%)	25 (48.6%)	1.0	1.0
-IPRE, preterm	23 (72.1%)	9 (27.9%)	0.21, 0.41 (0.10, 1.68)	0.24, 0.42 (0.10, 1.83)
Age at death (in days):				
-N (median, IQR)	50 [0.0 d; (0;30)]	34 (1.0 d (0;3)]	χ2 = 2.12 (P = 0.13)†	0.72, 0.94 (0.68, 1.31)
Illness severity at illness onset:				
-Mild/moderate (reference)	14 (53.5%)	12 (46.5%)	1.0	1.0
-Severe	34 (61.9%)	21 (38.1%)	0.54, 0.71 (0.23, 2.17)	0.66, 0.78 (0.25, 2.40)
**Late neonatal deaths (days 7-27)**				
Cause of death:				
-All other causes (reference)	0 (0.0%)	2 (100%)	NE	NE
-IPRE, preterm	5 (18.8%)	23 (81.2%)	NE	NE
Age at death (in days):				
N [median (IQR)]	4 [14.0 d (10;15)]	19 [12.0 d (8;18)]	χ2 = 0.95 (P = 0.33)†	0.40, 1.08 (0.89, 1.30)
Illness severity at illness onset:				
-Mild/moderate (reference)	3 (14.5%)	19 (85.5%)	1.0	1.0
-Severe	1 (13.3%)	5 (86.7%)	0.94, 1.13 (0.7, 17.2)	0.84, 1.10 (0.07, 16.49)
**All neonatal deaths (days 0-27):**				
Cause of death:				
-All other causes (reference	33 (42.1%)	45 (57.9%)	1.0	1.0
-IPRE, preterm	24 (69.2%)	11 (30.8%)	0.075, 0.32 (0.10, 1.12)	0.35 0.53 (0.14, 2.03)
Age at death (in days):				
N (median, IQR)	54 [1. 0 d (0;3)]	53 [3.0 d (1;10)]	χ2 = 20.3 (P < 0.001)†	0.008 1.15 (1.04, 1.27)
Illness severity at illness onset:				
-Mild/moderate (reference)	17 (38.7%)	27 61.3%)		
-Severe	37 (58.1%)	27 (49.9%)	0.10 0.26 (0.18, 1.16)	0.91 0.95 (0.35, 2.55)

NE – not estimable, OR – odds ratio, CI – confidence interval, IQR – interquartile range, AOR – adjusted odds ratio, IPRE – intrapartum related events

*Logistic regression models predicting any care provision or care seeking among neonatal deaths, adjusted for perceived illness severity at onset (severe vs mild/moderate) and age at death, in days.

†Kruskal-Wallis equality-of-populations rank test.

Among the deceased 1-59 month-olds, there was a nearly significant trend for formal health care to be more likely to be sought for those who died of infections compared to their counterparts who died of all other causes (AOR = 2.16, 95% CI = 0.97, 4.83; *P* = 0.059), when adjusted for illness severity and age at death (data not shown). However, no matter the cause of death, care was significantly less likely to be sought for 1-11 month-old children whose illness was perceived to be severe, compared to their counterparts with mild/moderate illness (AOR = 0.18; 95% CI = 0.06, 0.53; *P* = 0.002), though this was not true for 12-59 month-olds (AOR = 0.69; 95% CI = 0.25, 1.93; *P* = 0.48). This effect diminished with age, as the likelihood of careseeking for 1-11 month-olds increased for each additional month of age (AOR = 1.30; 95% CI = 1.07, 1.58; *P* = 0.009).

## DISCUSSION

The VASA study was conducted to provide essential information on the causes and social determinants of under-5 deaths, including neonates and 1-59 month-olds, in Tanzania. Most of the deceased children under-5 lived in households with precarious socio-economic conditions, lacking basic commodities such as electricity, sanitation and clean water, conditions that jeopardize child survival [15].

The age distributions of neonatal and child deaths were in-line with expectations, with both groups highly skewed toward younger ages [16]. Similarly, the excess mortality of male neonates was expected. The excess mortality of male neonates refers to the male disadvantage in infant mortality and suggests that male sex is more vulnerable to mortality [17,18], though the underlying mechanism has yet to be elucidated. Some prior studies have incriminated genetic and endocrine causes that might hamper pulmonary biomechanics and vascular development, leading to increased respiratory and neurological morbidity among preterm male neonates [19]. However, contrary to expectations created by the sex ratio usually being reported for children under-5 [20], there were more female than male deaths of 1-59 month-olds, which can be explained by their excess child mortality between ages one and five (4q1) [5,21].

The high percent of early (89%) and all neonatal (39%) deaths as a portion of all neonatal and under-5 deaths, respectively, contrasts with other countries in the region. In our Nigeria VASA study of under-5 deaths from 2008 to 2013, early neonates and neonates were 74% and 26% of all neonatal and under-5 deaths, respectively [22]; and in all sub-Saharan Africa during the Tanzania VASA study period, neonates were 30% of under-5 deaths in 2010, 34% in 2013, and 36% in 2016 [23-25]. This suggests that Tanzania is in the early stage of an epidemiological transition, with a larger proportion of deaths at the youngest ages due to relatively greater success in tackling infectious diseases of older children.

The most common causes of neonatal death were severe infection, IPRE and preterm delivery. The VASA study’s directly estimated preterm death proportion of all neonatal deaths (13.8%) is lower than the 2015 World Health Organization (WHO) modeled estimate (25.1%) for Tanzania [3]. Nevertheless, our estimate includes nearly all the 17.6% of neonatal deaths in our sample with pregnancy duration below eight months, calling into question the modeled estimate. Including the 10 IPRE deaths with comorbid preterm increases deaths with prematurity to 18.2%, including the one RDS death with pregnancy duration of eight months. Some VA hierarchies place preterm higher up, above IPRE [26,27], than does our hierarchy, contending that birth asphyxia resulting in hypoxic-ischemic encephalopathy (HIE) occurs only in full term newborns [28]. However, evidence shows that birth asphyxia and HIE do occur in premature infants [29], supporting our placement of IPRE above preterm. Displaying the IPRE deaths with comorbid preterm, as in **Figure 1**, Panel A, provides a fuller picture than single-cause preterm and IPRE attributions.

Other VASA estimates of neonatal causes of death varied in their correspondence with the 2015 WHO modeled cause estimates. The VASA and WHO IPRE proportions were nearly equal, 26.5% and 28.9%, respectively; while those for sepsis/meningitis/tetanus were more disparate, 13.9% and 20.5%, respectively and those for neonatal pneumonia (VASA 20.7%, WHO 6.4%) and congenital malformations (VASA 4.8%, WHO 12.6%) differed considerably, with the VASA estimates appearing more plausible.

The VASA direct and WHO modeled estimates of two of three leading causes of 11-59 month-old deaths, pneumonia (VASA 16.8%, WHO 20.0%) and diarrhea (VASA 15.2%, WHO 13.2%), were similar to each other, while the malaria proportions (VASA 14.0%, WHO 8.3%) differed substantially. The VASA and WHO proportions for some other causes, such as AIDS (VASA 6.3%, WHO 4.6%) were similar, while those for meningitis (VASA 6.9%, WHO 2.0%) and injury (VASA 7.4%, WHO 13.2%) differed considerably. Neither set of estimates has been validated, but the VASA estimates are derived from a validated analysis method, and previous work has shown that in some instances direct estimates may be preferable [8,22].

These comparisons of VASA and WHO estimates need to take into account that the WHO model excludes all its base VA deaths with an unspecified cause before presenting the deaths to national covariates, thus eliminating unspecified as a possible model cause. Similarly expunging unspecified from the EAVA causes would increase the level of each specific cause by an amount equal to its proportional share of the unspecified. This would bring some VASA and WHO estimates closer together and move some further apart. For example, proportionally allocating the unspecified neonatal deaths to neonatal sepsis/meningitis/tetanus would increase its proportion to 16.2%, moving it closer to the WHO estimate; while the diarrhea proportion of 11-59 month-olds would increase to 17.3%, moving it further from the WHO diarrhea estimate.

The VASA study found high coverage for most indicators along the continuum of care for newborns and 1-59 month-olds, indicating good access to primary health care facilities [30]. Health sector reforms have been instrumental in expanding access to health services in Tanzania. Indeed, the country has strived to implement maternal, newborn and child health programs in the post-MDG era to ensure sustained improvement in child survival and longevity of life [31]. Most importantly, the country undertook measures such as improving antenatal care attendance [32], skilled delivery care [33,34] and postnatal care [32] that would potentially have substantial positive impacts on neonatal and under-5 mortality.

However, the study also identified low coverage of other key interventions along the continuum of care, including early initiation of breastfeeding and hygienic cord care. If practiced routinely at both community and facility levels, these two interventions, together with proper thermal care, could reduce newborn deaths by up to 30% [35].

Only one-third of the deceased children were fully immunized against each of the six major preventable childhood diseases before they reached their first birthday [36]. This is surprising, as Tanzania’s immunization program is said to be one of the best performing in Africa, with almost 90% of 12-23 month-olds completing three doses of DTP-HepB-Hib vaccine, according to the 2015-16 TDHS-MIS [5]. The low completion rate found by the VASA study suggests a high proportion of drop-outs among the children who died. The reason for the low OPV1-3 rates compared to surviving 12-23 month-olds [37] needs to be further investigated. Previously, high dropout between early and final doses of the primary vaccine series have been shown to indicate health system barriers to attendance, failure to educate mothers of the need to return, or inadequate tracking of children registered at the health facility [36].

We have shown in the study a strong association between place of birth and neonatal death, and a higher percentage of health facility-based births (66%) and neonatal deaths (63%) than in previous VASA studies of under-5 deaths in Niger (28% and 19%), Cameroon (39% and 34%), Malawi (68% and 55%) and Nigeria (36% and 27%)[38]. In addition, nearly half the newborns died in the health facility where they were delivered, without leaving, most often from IPRE, severe infection and prematurity. This can be expected in settings such as Tanzania’s, with good access to institutional delivery care that is likely to encourage careseeking for maternal complications that result in neonatal death [39]. These findings suggest that, as Tanzania is in the early stage of an epidemiological transition, there is need for increased focus of its health system strengthening efforts on institutionally-based interventions targeting maternal labor and delivery complications and neonatal causes of death.

Severe infection was the most common cause of the community neonatal deaths, followed by preterm delivery and IPRE, emphasizing the need for a broad community intervention package including neonatal resuscitation, improved maternal recognition of newborn illness signs and early careseeking before the child becomes severely ill [40]. Tanzania was one of three countries to undertake a national scale-up of neonatal resuscitation in the “Helping Babies Breathe” initiative, with strong national leadership and an evaluation showing significant reduction in neonatal mortality, but not in some rural settings [41]. Scaling up this high-impact intervention to increase coverage in rural settings as well could boost Tanzania’s progress toward decreasing neonatal mortality.

In many instances, careseeking from home was lower for newborns who died from IPRE or prematurity than other causes. However, given that younger age was also associated with lower levels of careseeking, and that careseeking increased significantly for each additional day of age no matter the eventual cause of death, it appears that age rather than cause of death preempted careseeking from home. . This could suggest that issues such as concern for the child being too frail to travel, or the rapidity of death may have influenced careseeking for the youngest newborns more than poor recognition of danger signs, as suggested by other authors [42].

In the group of 1-59 month-old children, perceived severity of illness was negatively associated with formal careseeking, and the increased likelihood of seeking formal care for neonates as they grew older extended to 1-11 month-olds. In a study in rural Ethiopia, the authors found that perceived severity did not necessarily trigger care-seeking for neonates [43]. This contrasts with findings from studies conducted in Malawi [44], and Kenya [45]. Nonetheless, construct of the variable denoting illness severity that relies on caregivers’ perception has previously been contested [45], as this may require technical expertise especially if measurements are to be robust, standard and easily comparable. In any event, implementation of integrated Community Case Management (iCCM) of common childhood illnesses has yielded positive results in increasing access to and improving quality of essential child health services elsewhere [46]. It may be worthwhile for Tanzania to consider a scale-up of iCCM given our study findings that health care seeking is still suboptimal for the youngest neonates and children perceived as severely ill at onset [47].

While formal health care was sought for most 1-59 month-olds, the delay to seeking formal care was significantly lengthened among those who sought both informal and formal care, suggesting that seeking informal care caused the delay. Any barriers to formal careseeking resulting in even minor delays can be fatal, as child illness can progress rapidly and reduce the likelihood of surviving.

The overall high rate of careseeking for 1-59 month-olds was also tempered by a high rate of discharge and low referral from the first health provider. Multiple studies have commented on similar findings, emphasizing that attention needs to be directed to modifiable factors at the facility level [48-51]. Also, of concern in our findings is that many of the non-referred children had moderate-to-severe illness. In a case series, Font et. al investigated referral practices in rural Tanzania and found that too few children were referred to another provider [52]. Poor referral systems have also been flagged as a significant impediment to timely, safe and quality caesarean section deliveries for Tanzanian mothers with resultant poor health outcomes for their infants [53] [54]. It’s important to further understand the reasons for this low referral rate, as this could be interpreted as a health system failure in identifying critically ill children who need referral, or that health providers tend to refer children who have the means and social support to travel to the referral center [52].

### Limitations

This study has some limitations that were partly discussed in previous papers [12,55]. The long recall period of up to 7.5 years (range 1-7.5) may have impeded respondents’ recall of events and, thereby, the validity of the findings. This long recall was due to the retrospective design of the VASA studies required to include an adequate sample size of deaths. Given that and the fact that the respondents were the main caregivers of the deceased newborns, it is possible that the data may have been affected by different types of biases, including recall bias of past events and the likelihood of providing socially desirable answers to sensitive questions [11]. Nevertheless, the conversational and prompting modes used during the face-to-face interviews, along with the quality of the interviewers, supervisors and trainers, may have lessened the impact of these potential biases.

Limitations to the validity of verbal autopsies have been previously described [56]. Lastly, the study’s ability to identify risk factors for mortality was limited by not including a comparison group of survivors. On the other hand, all interventions examined by SA studies have been shown to be efficacious and effective in promoting newborn and child survival and, thus, included among the interventions examined by the Lives Saved tool [10] or recommended by the WHO. Therefore, these interventions should be accessible to all children.

## CONCLUSIONS

Tanzania has made good progress in child survival. Our study points to some key achievements and to areas of needed improvement. High coverage of most indicators along the continuum of care for newborns and young children indicates good access to primary health care facilities. Yet the country needs to address the gaps in coverage of early initiation of breastfeeding and hygienic cord care. The findings also suggest that Tanzania is in the early stage of an epidemiological transition, necessitating increased focus of its health system strengthening efforts on facility-based interventions targeting maternal labor and delivery complications and neonatal causes of death.

A large proportion of neonatal deaths still occur in the community, and Tanzania could make rapid progress by scaling up key interventions for neonates and promoting maternal recognition of neonatal illnesses and careseeking before the child becomes severely ill. The iCCM approach should be expanded to the whole country for its positive effect on overall use of health services at facility level. The time has come for Tanzania to complete the unfinished agenda in child survival.

## Additional material

Online Supplementary Document
