# Supplementary material for: Verbal/social autopsy analysis of causes and determinants of under-5 mortality in Tanzania from 2010 to 2016
Source: J Glob Health. 2020 Nov 8;10(2):020901. doi: 10.7189/jogh.10.020901 (PMC7699006; doi:10.7189/jogh.10.020901)
Supplement: Online Supplementary Document [file jogh-10-020901-s001.pdf]

Appendix S1. Neonatal and 1-59 month verbal autopsy algorithms and hierarchies

Neonatal algorithms and hierarchy

Neonatal tetanus
Age 3-27 days at death AND convulsions or spasms
AND either
((able to suckle normally during the first day of life AND stopped being able to suckle normally)
OR
(cried within 5 minutes after birth AND stopped being able to cry))

Congenital malformation
Head size very small at birth OR head size very large at birth OR mass defect on back of head or spine OR other severe abnormality (other reported severe abnormalities included in the algorithm: hydrocephalus, cleft palate, born with intestines outside the abdomen, born with no neck)

Intrapartum-related events (IPRE)
Birth asphyxia OR birth injury

Birth asphyxia
[Neonatal respiratory depression]: did not cry within 5 minutes after birth OR did not breathe immediately after birth
AND
(([Neonatal encephalopathy]: not able to suckle normally on the first day of life OR convulsions/spasms OR lethargy) OR 0 days old at death)

Birth injury
Bruises or signs of injury on the baby's body at birth

Preterm delivery
Pregnancy duration less than 9 months with Respiratory Distress Syndrome (RDS) OR pregnancy duration less than 8 months

RDS
Fast breathing starting on day 0 AND no fever AND no cold to touch

Meningitis
Fever AND bulging fontanelle or convulsions AND lethargic or unresponsive/unconscious

Diarrhea
More frequent loose or liquid stools than usual AND more than 4 stools on the day diarrhea was most frequent

Pneumonia
(Fast breathing lasting 1 day or more OR difficult breathing lasting 1 day or more and lasting until death) AND
2 or more of the following signs: lower chest wall indrawing, grunting, no or weak cry

Possible diarrhea

More frequent loose or liquid stools than usual AND VA sepsis AND no VA diarrhea

Possible pneumonia
Difficult breathing AND VA sepsis AND no VA pneumonia

Sepsis
Fever or cold to touch
OR
more than 1 of the following signs: fever or cold to touch, no normal suckling on the first day of life or stopped suckling, convulsions, vomited everything, stopped crying, lethargic or unconscious, lower chest wall indrawing or grunting

Neonatal jaundice
Yellow skin or yellow eyes AND (stopped being able to suckle normally OR lethargic OR unresponsive/unconscious) AND no fever or hypothermia

Hemorrhagic disease of the newborn
Bleeding from anywhere AND no fever or hypothermia

SUID (Sudden Unexplained Infant Death)
Died suddenly without appearing ill AND all illness signs/symptoms are negative

Neonatal hierarchy
Neonatal tetanus
Congenital malformation
IPRE (Intrapartum-related events)
Preterm delivery
Meningitis
Diarrhea
Pneumonia
Possible diarrhea (combined with diarrhea for final proportion)
Possible pneumonia (combined with pneumonia for final proportion)
Sepsis
Neonatal jaundice (part of Other)
Neonatal hemorrhage (part of Other)
SUID (part of Other)
Unspecified

1-59 month algorithms and hierarchy

Intrapartum-related event (IPRE)
Birth asphyxia OR birth injury

Birth asphyxia
[Neonatal respiratory depression]: did not cry within 5 minutes after birth OR did not breathe immediately after birth
AND
(([Neonatal encephalopathy]: not able to suckle normally on the first day of life OR convulsions/spasms OR lethargy) OR 0 days old at death)

Birth injury

Bruises or signs of injury on the baby's body at birth

Congenital malformation
Head size very small at birth OR head size very large at birth OR mass defect on back of head or spine OR other severe abnormality (other reported severe abnormalities included in the algorithm: hydrocephalus, cleft palate)

Injury
(Suffered from motor vehicle accident, fall, drowning, poisoning, venomous bite/sting, burn, violence or other injury AND death 1 day or less after the injury AND illness lasted 1 day or less)
OR
(suffered from motor vehicle accident, fall, drowning, poisoning, venomous bite/sting, burn, violence or other injury AND no other VA diagnosis [except malnutrition allowed])
OR
(suffered from motor vehicle accident, fall, drowning, poisoning, venomous bite/sting, burn, violence or other injury AND the injury was the first illness sign/symptom that occurred AND VA sepsis or fever)

AIDS
(Child with HIV/AIDS before the fatal illness started OR maternal positive HIV test or AIDS diagnosis)
OR
((swelling in armpits or whitish rash in mouth) AND (3 or more of the following: thin limbs, protruding belly, liquid stools more than 30 days, fever more than 30 days, skin rash more than 30 days, fast breathing, lower chest wall indrawing))

Malnutrition (underlying)
(Limbs became very thin during the fatal illness OR had swollen legs or feet during the illness) AND 1 of these was the first symptom of the illness

Measles
Child's age was 120 days or more AND rash that lasted 3 days or more AND fever that lasted 3 days or more AND the rash started on the face

Meningitis/encephalitis
Fever AND (stiff neck OR bulging fontanelle)

Dysentery
(More frequent loose or liquid stools than usual AND 5 or more stools on the day with most stools AND blood in stools)
OR
(more frequent loose or liquid stools than usual for more than 14 days AND blood in stools)

Diarrhea
(More frequent loose or liquid stools than usual AND 5 or more stools on the day with most stools AND no blood in stools)
OR
(more frequent loose or liquid stools than usual for more than 14 days AND no blood in stools)

Pertussis
Cough for more than 14 days AND either (severe cough, vomited after coughing, or stridor)

Pneumonia

(Cough for more than 2 days OR difficult breathing for more than 2 days) AND (fast breathing for more than 2 days OR lower chest wall indrawing OR grunting)

Malaria
Regional microscopic average parasite prevalence greater than 2%
AND either
((fever that continued until death and was on and off AND no stiff neck AND no bulging fontanelle) AND (pallor, difficult breathing, convulsions, or unconscious till death)
OR
(fever that continued until death and was severe AND no stiff neck AND no bulging fontanelle) AND (pallor, convulsions, or unconscious till death))

Possible dysentery
Loose or liquid stools AND (fever OR convulsions OR unconscious up till death) AND blood in the loose/liquid stools AND no VA dysentery

Possible diarrhea
Loose or liquid stools AND (fever OR convulsions OR unconscious up till death) AND no blood in the loose/liquid stools AND no VA diarrhea

Possible ARI
(Cough OR difficult breathing OR (fast breathing AND (lower chest wall indrawing, stridor, grunting or wheezing)))
AND
(severe cough or post-tussive vomiting or fast breathing or lower chest wall indrawing or grunting or stridor or wheezing or fever or convulsions or unconscious up till death)
AND
no VA Pertussis AND no VA pneumonia

Hemorrhagic fever
Fever AND either (bled from anywhere OR had areas of the skin that turned black)

Other serious infection
Fever AND (rash on trunk, abdomen or everywhere OR convulsions OR unconscious up till death) AND no VA malaria

Possible malaria
Regional microscopic average parasite prevalence greater than 2% AND VA Residual infection AND slept under a bed net sometimes or never

Residual infection
Fever and no other VA infectious diagnoses

Malnutrition (residual)
Limbs became very thin during the fatal illness OR had swollen legs or feet during the illness

Preterm delivery
Pregnancy duration less than 9 months with Respiratory Distress Syndrome (RDS) OR pregnancy duration less than 8 months

RDS
Fast breathing starting on day 0 AND no fever AND no cold to touch

1-59 month hierarchy

IPRE (Intrapartum-related events)
Congenital malformation
Injury
AIDS
Malnutrition (underlying: combined with residual malnutrition for final proportion)
Measles
Meningitis
Dysentery
Diarrhea
Pertussis
Pneumonia
Malaria
Possible dysentery (combined with dysentery for final proportion)
Possible diarrhea (combined with diarrhea for final proportion)
Possible ARI (combined with pneumonia for final proportion)
Hemorrhagic fever (part of Other infections)
Other serious infection (part of Other infections)
Possible malaria (combined with malaria for final proportion)
Residual infection (part of Other infections)
Malnutrition (residual: combined with underlying malnutrition for final proportion)
Preterm delivery
Unspecified
